# Aging-related changes in the gene expression profile of human lungs

**DOI:** 10.18632/aging.103885

**Published:** 2020-11-09

**Authors:** Ina Jeong, Jae-Hyun Lim, Jin-Soo Park, Yeon-Mok Oh

**Affiliations:** 1 Department of Pulmonary and Critical Care Medicine, National Medical Center, Seoul 05464, Republic of Korea; 2 Daechung Hospital, Daejeon 35403, Republic of Korea; 3 Asan Institute for Life and Science, Asan Medical Center, University of Ulsan College of Medicine, Seoul 05535, Republic of Korea; 4 Department of Pulmonary and Critical Care Medicine, and Clinical Research Center for Chronic Obstructive Airway Diseases, Asan Medical Center, University of Ulsan College of Medicine, Seoul 05535, Republic of Korea

**Keywords:** lung, aging, gene analysis, RNA-seq, transcriptome

## Abstract

Aging is a multifactorial process that leads to molecular and cellular changes, contributing to the susceptibility of most lung diseases. However, the molecular and genetic mechanism of lung aging remains poorly understood. Here, we performed RNA-seq transcriptome analysis of the lung tissues of 68 subjects and analyzed their gene expression profile to evaluate candidate genes related to lung aging. The subjects were classified into two groups (Younger group and Older group) based on their age. Lung tissues were obtained from surgically resected specimens, processed, and analyzed with RNA-seq. The median age of the subjects was 45 years in the Younger group and 74 years in the Older group. Around 71% and 53% of the subjects were female in the Younger and Older groups, respectively. After gene quality control and filtering, differentially expressed gene analysis showed that *MAP3K15, CHRM2,* and *GALNT13* were upregulated in the Younger group, whereas *COL17A1* and *EDA2R* were upregulated in the Older group. Multivariate analysis with adjustment for covariates showed that *EDA2R* was a risk factor for lung aging. Our study identified differences in the gene expression of the lungs of older subjects compared with younger subjects. These findings may have implications in lung aging.

## INTRODUCTION

With the large increase in life expectancy, population aging has rapidly become a major issue worldwide. Aging is one of the most well-known risk factors for many diseases and has been associated with increases in morbidity and mortality in various lung diseases [1]. The incidence and severity of chronic lung diseases such as chronic obstructive pulmonary disease (COPD), idiopathic pulmonary fibrosis (IPF), and lung cancer increase with age [1, 2]. In addition, the prevalence of acute lung diseases such as acute respiratory distress syndrome (ARDS) and pneumonia increases with age [1]. It is well known that measures of lung function such as vital capacity or diffusing capacity are reduced with aging, as observed in COPD patients in Global Initiative for Chronic Obstructive Lung Disease (GOLD) stage I [3]. In order to understand the decline in lung function with aging, it is necessary to elucidate the pathophysiology of lung aging. The physiologic aging of the lungs is known to be associated with the dilation of alveoli including enlarged airspace and decreased gas exchange surface area along with the loss of supporting tissues for peripheral airways, resulting in decreased elasticity and increased residual volume and functional residual capacity [3]. In addition to these emphysema-like structural changes in the lungs, respiratory muscle strength also decreases with aging due to intrinsic functional changes in the muscle [4]. Changes in the spine and ribs with aging can also affect normal lung function [4]. With aging, there is a decreased ability to clear mucus from the lungs due to reduced cough strength and alterations in the body’s ability to clear particles in the airways [4].

In terms of molecular and cellular changes, the nine hallmarks of aging have been proposed in a landmark paper [5], i.e., genomic instability, telomere shortening, epigenetic alterations, loss of proteostasis, dysregulated nutrient sensing, mitochondrial dysfunction, cellular senescence, altered intercellular communication, and stem cell exhaustion. Notably, inflammaging, which describes age-related low-grade chronic inflammation and immunosenescence, is often suggested as an additional conceptual hallmark of aging and identified and investigated as an independent conceptual entity in aging biology [6]. Senescence of the immune system in elderly individuals has been linked to many complex changes resulting in systemic immune dysfunction in both the innate and adaptive immune systems, which can increase susceptibility to infections [4, 7, 8]. Therefore, features related to aging may be related to chronic lung diseases such as COPD, lung cancer, and IPF with different degrees of activity, and there may be distinct aging-related characteristics in the pathophysiology of each chronic lung disease. At present, with the evolution of genomic technologies especially high throughput technologies, a large number of human tissue age-gene expression association studies have been conducted [9]. Accumulating evidence suggests that accelerated aging processes are major features of COPD [8, 10, 11]. One study reported that the downregulated aging gene signature in the lungs showed the most significant enrichment in genes associated with COPD-related biomarkers and pulmonary function [9]. Despite these findings, the genomic understanding of aging in normal lungs is still insufficient. In this study, we identified aging gene signatures in normal lung tissues and examined their functional characteristics to understand the molecular pathophysiology of lung aging by investigating the differences in gene expression during aging through RNA-seq.

## RESULTS

### Study subjects

Of the 68 subjects included in this study, 42 (61.8%) subjects were female (Table 1). The median age of the Younger and Older groups was 45 and 74 years, respectively. All subjects had quit smoking at least one month before lung resection. The number of subjects with past smoking history was higher in the Older group, and the amount of cigarette smoking was greater in the Older group than in the Younger group. Among the study subjects, the most common diagnosis was malignant carcinoma of the lungs (primary or metastatic) (88.2% of subjects in each group).

**Table 1:** Characteristics of subjects in the younger and older groups.

	**Younger (N = 34)**	**Older (N = 34)**
Age, years	45.0 ± 6.1	74.1 ± 2.9
Female gender, N (%)	24 (70.6%)	18 (52.9%)
BMI, kg/m^2^	22.2 ± 2.4	22.8 ± 2.8
Nonsmoker, N (%)	22 (64.7%)	18 (52.9%)
Past smoker, N (%)	12 (35.3%)	16 (47.1%)
Smoking amount, pack-years	15.3 ± 8.3 (N = 12)	39.1 ± 11.9 (N = 16)
Lung function		
FVC, % of predicted value	88.6 ± 4.8	85.1 ± 4.4
FEV1, % of predicted value	87.3 ±5.1	92.3 ± 4.7
FEV1/FVC, %	82 ± 5.0	76 ± 4.5
DLCO, % of predicted value	79.2 ± 9.1 (N = 26)	83 ± 10.6 (N = 18)

Data are presented as the mean ± standard deviation unless specified otherwise.

Abbreviations: N, number of subjects; BMI, body mass index; FVC, forced vital capacity; FEV1, forced expiratory volume in one second; DLCO, diffusing capacity of the lungs for carbon monoxide.

### Gene analysis

**Figure 1:**
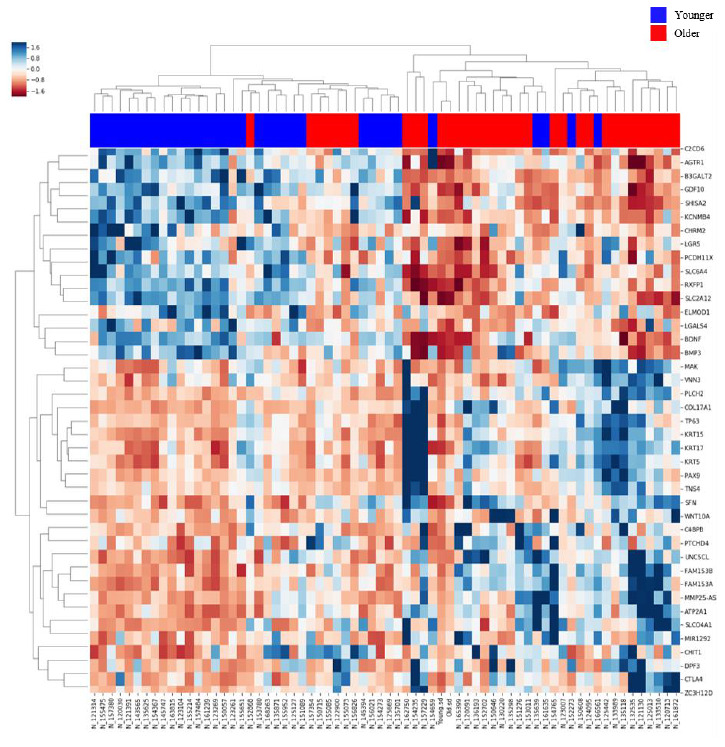
**Heatmap of gene expression in the lung tissues of the Older vs. Younger groups.** The heatmap of 80 genes with increased or decreased gene expression is illustrated with the hierarchical clustering of gene expression data for the Older and Younger groups. The colored column sidebar at the top indicates the status of the subjects (blue - Younger group; red - Older group). The information has been revised for better flow and readability. Please check if the revised information conveys your intended meaning.

Of 14,775 genes, 4,108 genes were differentially expressed between the age groups by Student’s t-test with p value < 0.01. After adjusting the p value using the false discovery rate (FDR) method, 2,442 genes were differentially expressed. Among these genes, 897 genes were upregulated in the Older group, and 1,545 genes were upregulated in the Younger group. We performed linear regression analysis to adjust for the effect of smoking, and 3,565 genes were differentially expressed between the two groups with p value < 0.01. Of these genes, only 158 genes were significantly associated with smoking rather than aging; thus, we used the former differential gene expression data by the t-test for further evaluation due to little effect of smoking on DEGs. Hierarchical clustering analysis was also performed, and a heatmap for the two groups was generated (Figure 1).

**Figure 2:**
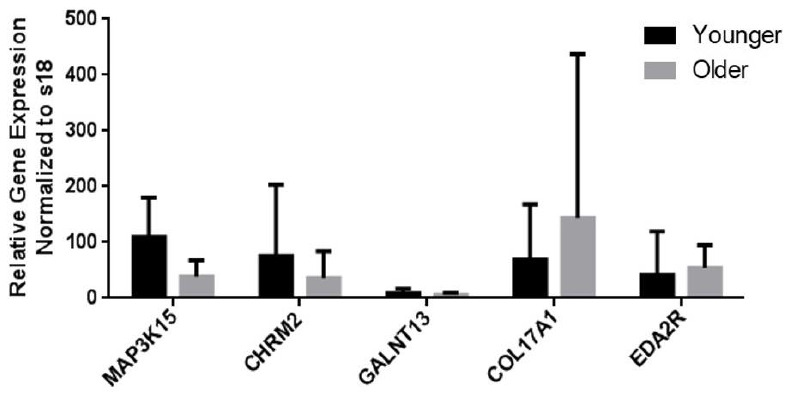
**mRNA expression levels of genes as measured by qRT-PCR analysis.** The levels of mRNA transcripts that encode aging-related marker genes are shown. Data are presented as the mean ± standard deviation (*N* = 32).

Among the differentially expressed genes (DEGs) with significance, the top 10 genes with the largest fold changes in both the Younger and Older groups are shown in Tables 2 and 3. Among them, genes that could be related to aging or the pathogenesis of lung disease were selected from each group based on literature review. *MAP3K15, CHRM2,* and *GALNT13* from the Younger group and *COL17A1, MUC16,* and *EDA2R* from the Older group were re-tested and validated by quantitative reverse transcription polymerase chain reaction (qRT-PCR) (Figure 2).

**Table 2:** Top 10 genes with increased expression (based on fold change) in the younger group compared with the Older group.

**Gene**	**Gene function**	**Fold change log2 [Older/Younger]**	**p value**	**Expression level in Younger (FPKM)**	**Expression level in Older (FPKM)**
*MAP3K15*	Mitogen-activated protein kinase kinase kinase 15	-0.99	4.02×10^−7^	0.79	0.40
*CHRM2*	Cholinergic receptor muscarinic 2	-0.92	1.52×10^−3^	0.69	0.37
*GALNT13*	Polypeptide N-acetylgalactosaminyltransferase 13	-0.89	2.75×10^−8^	0.98	0.53
*MATN3*	Matrilin 3	-0.87	1.99×10^−9^	2.89	1.58
*ELMOD1*	ELMO domain containing 1	-0.87	2.69×10^−4^	0.47	0.26
*SHISA2*	Shisa family member 2	-0.83	5.52×10^−6^	0.92	0.52
*FIBIN*	Fin bud initiation factor homolog (zebrafish)	-0.81	2.42×10^−8^	1.81	1.04
*B3GALT2*	Beta-1,3-galactosyltransferase 2	-0.80	3.13×10^−6^	0.67	0.38
*P2RY1*	Purinergic receptor P2Y1	-0.79	7.84×10^−9^	1.18	0.68
*SLC6A4*	Solute carrier family 6 member 4	-0.77	4.72×10^−6^	4.09	2.40

Abbreviation: FPKM, fragments per kilobase of transcript per million fragments mapped.

**Table 3:** Top 10 genes with increased expression (based on fold change) in the Older group compared with the younger group.

**Gene**	**Gene function**	**Fold change log2 (Older/Younger)**	**p value**	**Expression level in Younger (FPKM)**	**Expression level in Older (FPKM)**
*COL17A1*	Collagen type XVII alpha 1 chain	1.80	3.39×10^−7^	0.65	2.27
*MUC16*	Mucin 16, cell surface associated	1.60	1.60×10^−4^	0.61	1.84
*TNS4*	Tensin 4	1.48	1.27×10^−3^	0.32	0.90
*EDA2R*	Ectodysplasin A2 receptor	1.47	5.34×10^−10^	0.18	0.49
*PAX9*	Paired box 9	1.24	1.15×10^−3^	0.41	0.97
*KRT15*	Keratin 15	1.16	1.41×10^−6^	1.45	3.24
*FBXW10*	F-box and WD repeat domain containing 10	1.15	1.47×10^−4^	0.31	0.68
*FAM153A*	Family with sequence similarity 153 member A	1.13	2.69×10^−7^	0.97	2.13
*ZC3H12D*	Zinc finger CCCH-type containing 12D	1.09	5.39×10^−5^	0.21	0.44
*PTPRZ1*	Protein tyrosine phosphatase, receptor type Z1	1.09	2.98×10^−3^	0.37	0.80

Abbreviation: FPKM, fragments per kilobase of transcript per million fragments mapped.

We performed simple and multiple linear regression analysis on 5 DEGs that were consistently validated by qRT-PCR. In simple linear regression analysis, each of the 5 genes was positively or negatively associated with aging; however, in multiple linear regression analysis, only *EDA2R* was a significant contributing factor in the Older group (Supplementary Figure 1, Supplementary Table 2). The expression of *EDA2R* (p value = 2.77×10^−8^) was an independent risk factor in the Older group after additionally adjusting for history of smoking and gender. In addition to *EDA2R, GALNT13* (p value = 0.007) and history of smoking (p value = 0.008) were significant risk factors (Supplementary Table 3).

### Validation

For technical validation, qRT-PCR was performed to identify DEGs (Supplementary Table 1). The mRNA expression levels of 6 genes (Younger group vs. Older group) were similar to the results of RNA-seq analysis, except for *MUC16*. Therefore, *MUC16* was excluded in the additional analysis (Figure 2). We also performed qRT-PCR on two genes (*ACER2* and *CHRM3*), which have been found to increase in patients with COPD with/without emphysema in our previous study, and these genes showed no significant difference between the two groups in the validation.

## DISCUSSION

In this study, we identified several genes with increased expression in the Older group, and there were significant differences in the gene expression between the two groups according to the age. One of the genes, ectodysplasin A2 receptor (*EDA2R*), was recently reported as a strong candidate gene for aging [11]. According to that study, aging strongly affected gene expression in the lung tissues. *EDA2R* was also highly associated with aging in the adipose tissues, artery, heart, muscle, and skin tissues in the GTEx project, which evaluated the aging signature of these tissues [9]. In the present study, *EDA2R* was the most differentially expressed according to the p value in the Older group compared with the Younger group as well as the fourth most upregulated gene according to the fold change. Moreover, when multivariate linear regression analysis was performed, *EDA2R* was the only significantly different gene in DEG analysis. Although little is known about this gene, *EDA2R* belongs to the tumor necrosis factor receptor superfamily, which is involved in various signaling pathways. It is known to be associated with nuclear factor kappa B (NF-kB) and p53 signaling pathways and can promote apoptotic signaling through the binding of its ligand EDA-A2 [12].

Another gene with increased expression in the Older group, collagen type 17 alpha 1 (*COL17A1*), encodes the alpha chain of type XVII collagen, which is a major structural component of hemidesmosomes and plays an essential role in strengthening and stabilizing the skin. Mutations in this gene are associated with both generalized atrophic benign and junctional epidermolysis bullosa [13]. Recently, patients with *COL17A1* mutation have been reported to exhibit a premature aged skin phenotype, including hair and hair loss [14]. Interestingly, *COL17A1* was the only procollagen gene that was increased with aging among 8 procollagen genes, which were altered with aging in a study of mouse skeletal muscles [15]. However, the role of *COL17A1* in lung aging remains unknown and should be further evaluated. *Mucin 16* (*MUC16*) is known as ovarian cancer antigen CA-125. It was reported that CA-125 level could be increased in chronic medical conditions including cancer. [16, 17]. According to a lung transcriptome study, *MUC16* was one of the genes in a second large cluster that included dynein and other *MUC* genes, which are exclusive to the respiratory epithelium and goblet cells of bronchial structures [18]. In addition to their normal physiological role in protecting epithelial cells, mucins have been shown to participate in various diseases including cancer. Although *MUC16* showed increased expression in the Older group compared with the Younger group, qRT-PCR did not show consistent results; thus, we excluded *MUC16* from linear regression analysis.

Cholinergic receptor muscarinic 2 (*CHRM2*) was downregulated in the Older group compared with the Younger group. The *CHRM2* gene encodes the M2 muscarinic acetylcholine receptor and belongs to the superfamily of G protein-coupled receptors, which show functional diversity in various cellular responses via the binding of acetylcholine to these receptors [19]. Although, *CHRM2* is known to activate several signaling pathways in the nervous system, and little is known about the effect of this gene in pulmonary disease. Recent studies of the pathogenesis of allergies and asthma have reported that muscarinic receptors may modulate airway reactivity [19]. Certain types of *CHRM2* polymorphism may be associated with disease severity, lower lung function test values, frequent exacerbations, and poor response to anti-cholinergic drugs [20]. *CHRM2* has also been reported as a candidate gene for nicotinic addiction by modulating presynaptic auto-regulation in the cholinergic system [21]. Moreover, the targeted deletion of *CHRM2* showed significantly reduced hyperoxia-induced lung injury in a mouse experiment [19]. Nevertheless, further studies are needed to identify the role of *CHRM2* in modulating the response to hyperoxia. Polypeptide N-acetylgalactosaminyltransferase 13 (*GALNT13*) was another gene with decreased expression in the Older group compared with the Younger group. *GALNT13* belongs to the GalNAcT family of enzymes, which initiate the O-glycosylation of mucins. A previous study reported that the expression of *GALNT13* mRNA was the highest in brain tissues, and it may be a strong predictor of poor clinical outcome in neuroblastoma patients [22]. However, the role of *GALNT13* is unknown in terms of lung disease. A genome-wide interaction study on occupational exposures in relation to the level of lung function reported that *GALNT13* was one of the candidate genes that might be involved in biological pathways leading to lung function impairment [23].

It is well known that cigarette smoking could directly affect gene expression; however, gene expression differences between smokers and nonsmokers are largely reversible after smoking cessation [24]. As the possible effects of current smoking on gene expression can be relatively large, we included only past smokers who stopped smoking at least one month before surgery. This could help offset the effects of current smoking on gene expression. The results were not significantly different after we corrected for the potential confounder (smoking status). In addition, for the validation of differential gene expression, qRT-PCR of genes in the lung tissues was performed on 6 candidate genes, which showed similar results in 5 out of 6 genes.

Our study had some limitations. First, the total study population was 64; more data would be needed to verify candidate genes for aging. Second, the study had a retrospective nature as the study cohort was not originally designed for genetic analysis. The clinical features of the cohort who underwent lung resection might be not suitable for evaluating and comparing genetic differences between younger and older individuals. Moreover, the mean age of the Younger group was not too low but relatively lower (45 years in the Younger group compared with 72 years in the Older group). In addition, most patients had malignancy; however, it was in the early stage (stage I or II, 88.2%). This may be explained by the characteristics of the cohort, which consisted of patients who underwent surgery because of the presence of nodules in the lungs. However, the proportion of patients with malignancy was similar in both groups, and most patients (88%) who had malignancy remained at stage I or II. This could offset the effect of cancer on gene expression in the two groups.

In conclusion, we identified several genes that may be associated with normal lung aging by RNA-seq. *EDA2R* was an independent factor after confounder adjustment for subjects without chronic lung disease in the Older group. Further larger studies are needed to validate these results.

## MATERIALS AND METHODS

### Study subjects and specimen

The subjects were selected from a registered in-house tissue storage system (the Asan Biobank), in which lung tissues for this study had been stored from 2012 to 2016. The lungs were resected mostly due to the presence of tumors (either benign or malignant). Immediately after the resection, lung tissues were obtained at a site as far away as possible from the tumor tissues and stored under -170°C of vaporized nitrogen in the Asan Biobank. Subjects who had abnormal lung function before lung resection or any history of chronic lung diseases (asthma, COPD, interstitial lung disease (ILD), lungs destroyed by tuberculosis, or bronchiectasis) were excluded. Current smokers who smoked within one month before lung resection were also excluded. Subjects finally diagnosed as having a malignancy with TNM stage II or higher after lung resection were excluded. To compare differences in the gene expression of the lungs according to age, the subjects were classified into two groups based on their age: the Younger group and Older group. Among the subjects who met the inclusion criteria, we chose two contrasting groups consisting of younger subjects (Younger group) vs. older subjects (Older group) with an appropriate number of sample size in each group (see below). We excluded subjects with intermediate ages.

### Justification of sample size

The hypothesis of this study was that there would be a significant difference between the Younger and Older groups. Based on a level of significance of α = 0.05 and a power of 80% for detecting a difference in the expression level of a gene(s) of two times higher, the sample size per group was calculated to be 17. Considering additional adjustment for smoking and gender, the final sample size was determined as 34 per group with a total sample size of 68 for two groups.

### RNA preparation and sequencing

Total RNA was isolated from apparently normal fresh frozen lung tissues that were remote from the lung cancer. RNA integrity was assessed using an Agilent Bioanalyzer system, and RNA purity was assessed using a NanoDrop spectrophotometer. The total RNA (1 μg) was used to generate cDNA libraries with the TruSeq RNA Library Prep Kit. The protocol consisted of poly A-selected RNA extraction, RNA fragmentation, reverse transcription using random hexamer primers, and 101 bp paired-end sequencing using the Illumina HiSeq 2500 system.

### Quality control and data management

For quality control, read quality was verified using FastQC, and read alignment was verified using Picard. All samples had a Phred score higher than 20. To preprocess RNA-seq data, we removed the adapter sequence using Trimmomatic and removed reads with a Phred score below 15. UCSC hg19 human genome and transcriptome references were used to map the cDNA fragment obtained from preprocessing. We used bowtie2 aligner and HISAT2 to map reads and used StringTie to calculate the fragments per kilobase of transcript per million mapped reads (FPKM). We only used genes with a FPKM value above 0 in at least one sample. Further analysis was conducted on 14,775 out of 27,685 genes after filtering for genes with 0 counts in the whole samples, noncoding genes, and low-variance genes.

### qRT-PCR analysis

For technical validation, we re-tested some of the genes that were differentially expressed by qRT-PCR. Gene expression in the lung tissues was quantified by qRT-PCR using LightCycler 480 (Roche, Mannheim, Germany) with LightCycler 480 SYBR Green I Master (Roche, Mannheim, Germany). Total RNA was isolated using the RNeasy Plus Mini Kit (Qiagen, Valencia, CA, USA), and 1 μg of each sample was reverse-transcribed using the Maxima First Strand cDNA Synthesis Kit (Thermo Scientific, Waltham, MA, USA) for real-time qRT-PCR.

### Statistical analysis

Clinical statistical analyses were performed using SPSS v26.0 (SPSS; Chicago, IL, USA). We performed quantile normalization to adjust for between-sample bias using the preprocessCore R library. Linear regression was performed using Python StatsModels 0.10.2 to identify DEGs between the Younger and Older groups and correct the effects of smoking.

## Supplementary Material

Supplementary Figure 1

Supplementary Tables
