# Supplementary material for: Aging-related changes in the gene expression profile of human lungs
Source: Aging (Albany NY). 2020 Nov 9;12(21):21391–403. doi: 10.18632/aging.103885 (PMC7695411; doi:10.18632/aging.103885)
Supplement: Supplementary Figure 1 [file aging-12-103885-s001..pdf]

SUPPLEMENTARY TABLES

Supplementary Table 1. Primer sequences for qRT-PCR analysis.

Gene symbol	Forward	Reverse	Accession number
MAP3K15	CCTTCTACGACGCAGATGTTG	GCATCGGTGTCATGGTACAAGA	NM_001001671
CHRM2	AACTCCTCTAACAATAGCCTGGC	GTTCCCGATAATGGTCACCAAA	NM_000739
GALNT13	TTGCCCTTAATAGAAGTCTGCCA	TGGGGAACGATTTATCACACTG	NM_052917
COL17A1	ACCAGCAATGGCTATGCTAAAA	GCCTCGTGTGCTTCCAGTT	NM_000494
EDA2R	TCCAAGGATTGTGGTTATGGAGA	AGCACAGGTGATGCAACTCTG	NM_021783

Supplementary Table 2. Results of multiple linear regression analysis of 5 genes associated with aging .

Model Summary[b]

Model	R	R square	Adjusted R square	Standard error of the estimate	Durbin-Watson
1	0.743[a]	0.552	0.516	10.716	0.960

a. Predictors: (Constant), EDA2R, CHRM2, COL17A1, MAP3K15, GALNT13.
b. Dependent variable: Age.

ANOVA[a]

Model		Sum of squares	df	Mean square	F	Sig.
1	Regression	8783.068	5	1756.614	15.298	0.000[b]
	Residual	7119.402	62	114.829		
	Total	15902.471	67			

a. Dependent variable: Age.

b. Predictors: (Constant), EDA2R, CHRM2, COL17A1, MAP3K15, GALNT13.

Coefficients[a]

Model		Unstandardized coefficients		Standardized coefficients	t	Sig.	Collinearity statistics	
		B	Std. error	Beta			Tolerance	VIF
1	(Constant)	53.153	4.946		10.747	0.000		
	MAP3K15	0.021	4.733	0.001	0.004	0.997	0.504	1.984
	CHRM2	-1.822	2.736	-0.069	-0.666	0.508	0.676	1.479
	GALNT13	-6.203	4.538	-0.188	-1.367	0.177	0.380	2.628
	COL17A1	0.037	0.111	0.031	0.335	0.738	0.837	1.195
	EDA2R	43.459	8.211	0.581	5.293	0.000	0.600	1.668

a. Dependent variable: Age.

Abbreviation: VIF, Variance inflation factor; Std., Standard; Sig., Significance.

Supplementary Table 3. Results of multiple linear regression analysis of 5 genes associated with aging with adjustment for history of smoking and gender.

Model Summary[b]

Model	R	R square	Adjusted R square	Standard error of the estimate	Durbin-Watson
1	0.772[a]	0.596	0.549	10.342	1.140

a. Predictors: (Constant), EDA2R, CHRM2, COL17A1, MAP3K15, GALNT13, History of smoking, Gender.

b. Dependent variable: Age.

ANOVA[a]

Model		Sum of squares	df	Mean square	F	Sig.
1	Regression	9484.607	7	1354.944	12.667	0.000[b]
	Residual	6417.863	60	106.964		
	Total	15902.471	67			

a. Dependent variable: Age.

b. Predictors: (Constant), EDA2R, CHRM2, COL17A1, MAP3K15, GALNT13, History of smoking, Gender.

Coefficients[a]

Model		Unstandardized coefficients		Standardized coefficients	t	Sig.	Collinearity statistics	
		B	Std. error	Beta			Tolerance	VIF
1	(Constant)	57.028	5.978		9.539	0.000		
	MAP3K15	1.125	4.590	0.028	0.245	0.807	0.499	2.004
	CHRM2	-0.344	2.704	-0.013	-0.127	0.899	0.645	1.551
	GALNT13	-9.289	4.543	-0.282	-2.045	0.045	0.354	2.827
	COL17A1	-0.007	0.109	-0.006	-0.064	0.949	0.804	1.243
	EDA2R	46.831	8.081	0.626	5.795	0.000	0.577	1.734
	History of smoking	-7.636	3.667	-0.246	-2.082	0.042	0.483	2.070
	Gender	-0.879	3.625	-0.028	-0.242	0.809	0.507	1.973

a. Dependent variable: Age.

Abbreviation: VIF, Variance inflation factor; Std., Standard; Sig., Significance.
