# Supplementary material for: Aging-related changes in the gene expression profile of human lungs
Source: Aging (Albany NY). 2020 Nov 9;12(21):21391–403. doi: 10.18632/aging.103885 (PMC7695411; doi:10.18632/aging.103885)
Supplement: Supplementary Tables [file aging-12-103885-s002..pdf]

SUPPLEMENTARY FIGURES

Supplementary Figures

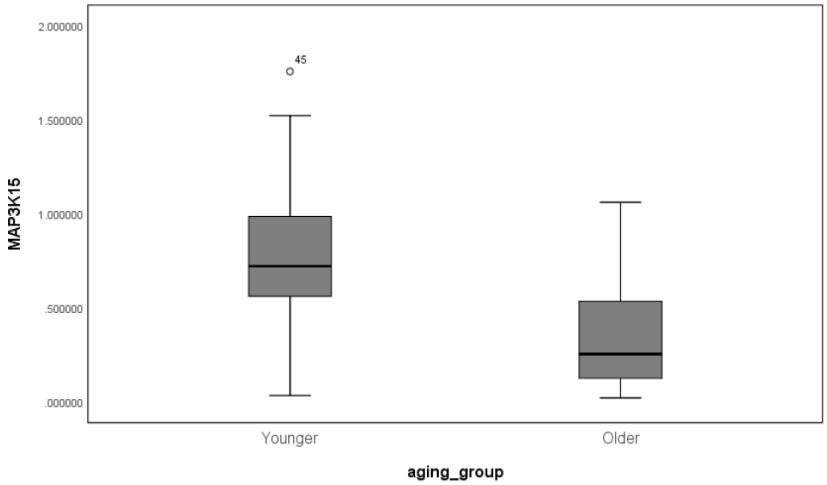

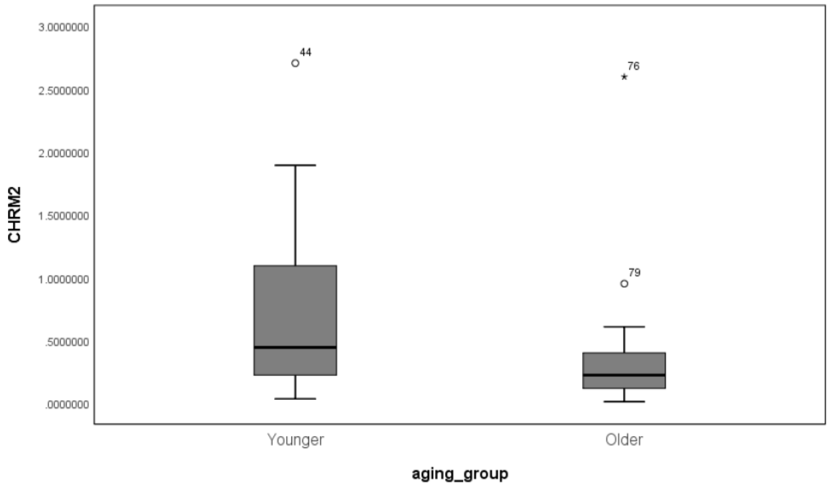

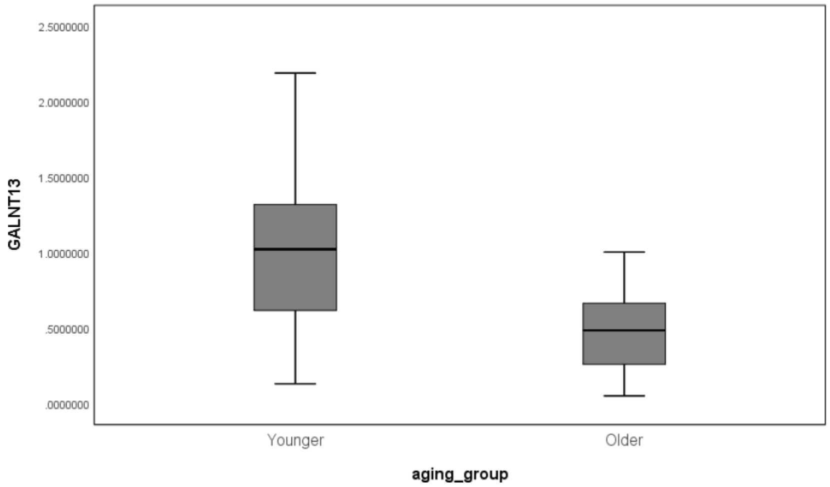

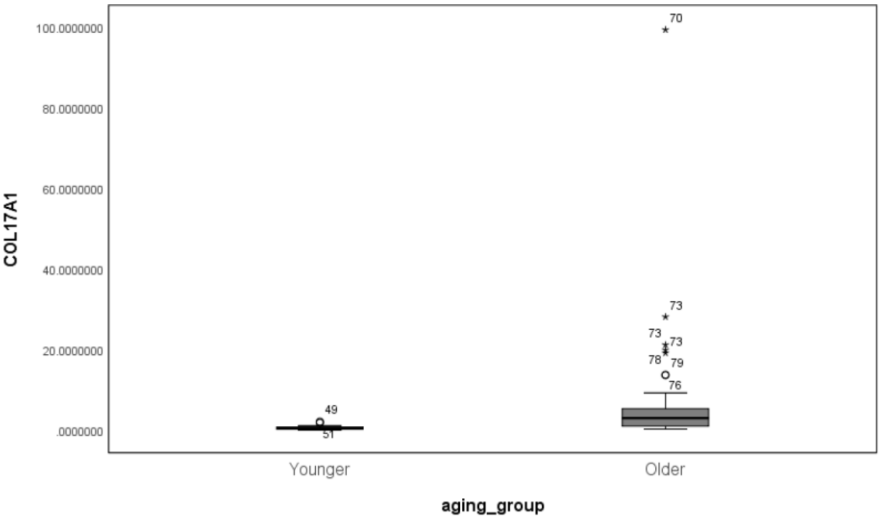

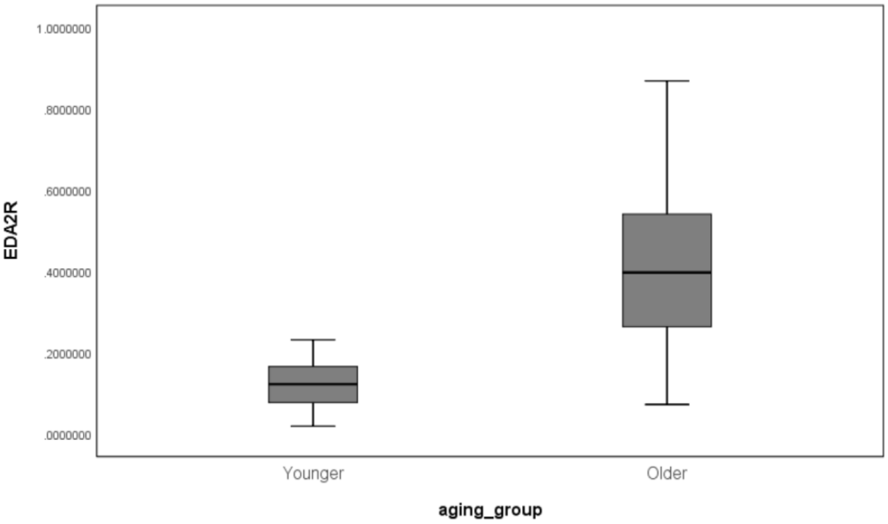

Supplementary Figure 1. Box plots of the expression profile (based on FPKM value) of the top 5 DEGs that showed significant differences in DEG analysis according to the age group.
